# Retrospect of the Late Improvements in Medicine, Surgery, &c.

**Published:** 1835-01-01

**Authors:** 


### ORIGINAL COMMUNICATIONS.

### RETROSPECT OF THE LATE IMPROVEMENTS IN MEDICINE, SURGERY, &c. By the Editor.

It is hardly necessary to premise that the short Retrospect which we are about to attempt makes no pretensions to be either a history of medicine, or a comparison of what has been done with what might have been done. We shall merely venture to execute the humble, but perhaps useful, office of pointing out a few of the more recent improvements in the art of healing, together with such discoveries in anatomy and physiology as may be interesting, either from settling some long-disputed point, or from their having some tendency to improve the practice of physic. We regret that we cannot commence this summary by announcing that a specific, or at any rate a tolerable method of treatment, has been discovered for the most frightful disease, the nearest approximation to a pestilential epidemic which has been known in this country for many generations: the Asiatic cholera, though less fatal in 1834 than in 1833, and less fatal in 1833 than in the previous year, seems scarcely to be under the influence of medicine. It would be endless to recount the various methods of treatment which have been tried since the first invasion of the epidemic. Laudanum and brandy, calomel with cold water, salt-and-water emetics, saline medicines, and that desperate resource—injection into the veins, have all been employed with a success which has amply satisfied a few patrons of each method, but has been extremely unsatisfactory to the profession at large. Now, this is not the case when any thing excellent, or good, or tolerable, is brought forwards. The use of iodine in bronchocele, of colchicum in gout, the substitution of the sulphate of quinine for bark in substance, leave many dissatisfied; but the real worth of these several remedies overwhelms opposition, and a few failures are not sufficient to condemn a medicine before so impartial a tribunal as the medical public.

It is therefore to be feared that the practitioners who have hitherto extolled various remedies as specifics in cholera, have been mistaken, not indeed as to the fact of their patients surviving, but as to the nature or intensity of the disease: for if any plan of treatment, that by calomel and cold water for instance, were gifted with the attributed efficacy, it is difficult to suppose that it would not spread. The neighbouring practitioners would be compelled, for very shame, to adopt the use of the specific, and their accumulated evidence daily increasing in quantity and respectability would bear down all opposition. There is one remedy which has been used in Germany with considerable success, and which has a very plausible theory in its favour. This remedy is the sulphate of quinine, and it is recommended from the obvious resemblance of the Asiatic cholera in many points to an ague of the greatest intensity. Our readers will find the particulars in our second number.

*Homœopathy*. We foresee that many will censure us for devoting a page to homœopathy in an essay on *improvements* in the practice of physic; and it may be objected, moreover, that we are performing a work of supererogation, as we have already explained the principles of infinitesimal medicine. To this we would answer, that a retrospect of this kind being chiefly intended for those who live remote from the great foci of knowledge, an account of any sweeping changes in the practice of physic, however dubious their merit, cannot be wholly uninteresting; and that a compendium of the subject may be advantageous to those who have neither time nor inclination to read a long review of the *Organon der Heilkunst*. The principles of homœopathy are two; the first is, that, to cure a disease, we must administer a remedy which would produce that disease in a healthy person: this rule requires of course to be understood liberally and indulgently, as it is not very easy to find a drug which will produce gout for instance, or scarlatina, in a healthy person. It seems sufficient therefore if the remedy will produce some of the more striking symptoms of the disease to be cured; thus, belladonna, say the homœopathists, will bring out an eruption like that of scarlatina, as well as some other symptoms of the disease, and therefore is its appropriate remedy. The second principle is, that medicines are to be given in doses infinitely small; a drop of tincture of belladonna is to be diluted with 100 drops of water, and a drop of this diluted solution is again to be diluted with 100 drops of water, and so on for 6, 8, or 10 times; and then a fraction of a drop of the last and weakest solution is the dose. So that there is no danger of the patient being poisoned.

It must be confessed that these principles have at first sight something rather ludicrous about them, and might have been mistaken for a new way of curing disease sent forth from the Academy of Laputa. Yet this theory, though strange and fantastic, deserves the attention of the most enlightened practitioner; partly because it is now put into practice by a host of continental physicians, and partly because it is an undoubted fact that myriads of patients who have taken nothing but these infinitesimals, these ghosts of departed medicines, as we once before called them, do nevertheless get well. How do they get well? Not, we think, by the decillionths of aconite or pulsatilla, but by the *vis medicatrix naturæ*. It is to this renovating power which resides in every unbroken constitution that the followers of Hahnemann owe their success, and it is to this that we would have the physician trust more frequently in those chronic cases where delay is less dangerous than mistake. Thus it would be better that a patient should take the infinitesimals appropriated to an enlarged liver, rather than pass through what Dr. Abercrombie calls "long and ruinous courses of mercury," especially if the existence of the said enlargement were rather dubious. There is a curious paper on the relative advantages of aconite and bleeding in the treatment of inflammatory diseases, which was read before the "Sociéte Homœopathique Gallicane," last autumn. The author does like to reject bleeding altogether, as homœopathy bids him, and therefore without giving up aconite, (the certain remedy, it seems, for inflammation,) he wishes to premise venesection. It will be objected, he says, that by thus joining a bit of the old and a bit of the new system of physic, it will not be known which ought to have the honour of the cure. This is of no importance, replies M. Chuit; the great point is to cure as quickly as you can, "and I care not, if allopathy says, it is the bleeding that has cured the patient; and homœopathy declares, that it is the aconite; the only object is the cure."

The *Bibliothèque Homœopathique*, to which we are indebted for Mr. Chuit's paper, contains extracts from a German journal devoted to the same system. They relate instances of homœopathic cures, some given at length, and others with a more than laconic brevity. Thus, "a black vomit (*melæna* 2) was completely cured in six hours by *ver.* $\frac{000}{\text{iv}}$." "Hromada says, that he has cured a tuberculous phthisis with *cortex sambuci* 3; he wishes this substance to be tried again, as well as *urtica urens*, a popular remedy in the same disease."

Let us not omit to add that Andral has tried Hahnemann's system at Paris, in a considerable number of cases, but it was found to fail entirely. The *Bulletin Médical Belge* for November, 1834, contains three cases of inflammatory disease treated homœopathically, by way of experiment. Aconite was the medicine given, but without success; and the increasing violence of the symptoms rendered the abstraction of blood necessary in each case.

*Functional Diseases of the Spine.* It may seem superfluous to touch upon this subject again after having given so long an analysis of Messrs. Griffin's work in this very number, but the topic is so important, and the improvement in practice so considerable, that an account of the recent progress of physic would be incomplete were it omitted. We must therefore stand excused for mentioning it again, and giving, as it were, a second distillation of the admirable Treatise in question. Dr. Griffin has found, by repeated experience, that a morbid irritability of the spinal marrow is attended by an increased sensibility of the structure which lie above it, and may thus easily be detected by pressure; and the train of symptoms depending on this irritability may, in most instances, be successfully treated by counter-irritation applied to the tender spot. The symptoms of course vary according to the part of the spine which is affected; the cervical, dorsal, and lumbar nerves, separately or in conjunction, having the power of stimulating the thoracic, abdominal, and peloric viscera, into diseased action, which is continually mistaken by hasty practitioners for the result of inflammation. Instances are given by Messrs. Griffin where blisters to the back relieved pain in the chest, when blisters applied to the chest itself were ineffectual.

*Belladonna in Hooping-cough.* The use of belladonna in hooping-cough is by no means new, but it has not been generally adopted; perhaps from practitioners having been disappointed in its effects when the drug was adulterated, and alarmed at its effects when the drug was genuine. Dr. Jackson, who has lately written a paper on this subject in the American Journal of the Medical Sciences, was at first baffled, owing to the falsification of the drug employed, which in some instances appears not to have been belladonna at all. The genuine extract of belladonna may easily be distinguished by its power of dilating the pupil; hyoscyamus, it is true, will do the same, but in a much less degree. Dr. Jackson's subsequent experiments with the genuine extract were perfectly successful; and he advises us to give it in doses of $\frac{1}{6}$ of a grain to infants three months old, every three hours from sunrise to sunset, until the pupil be dilated. If the extract is very good, we should conjecture that this dose would be too large.

*Iodine in Syphilis.* This plan of treatment has been adopted of late by many eminent practitioners; among others, by Mr. Tyrrell, who informs us that he is perfectly satisfied with the results, and that he has used it even for the primary symptoms with great success. We would warn our readers against the common error of giving iodine in too large doses; if this be avoided, it is one of the safest, as well as most useful remedies we possess. Perhaps the best method of administering it is to dissolve half a grain of iodine and one grain of the hydriodate of potass in eight ounces of distilled water; and this may be the daily dose.

*Discovery of the Itch Insect.* It has long been a subject of doubt whether an insect exists in the vesicles of the itch. This doubt has at length been cleared up by M. Renucci, an Italian student at Paris, who has succeeded in extracting the insect from the small epidermic canal terminating in the vesicle, in which the insect is generally lodged. It is sometimes, though rarely, situated in the vesicle itself. This discovery has been confirmed by a host of other observers; and M. Renucci is consequently entitled to the reward of 300 francs long since offered by M. Lugol.

*Antidote to Arsenic.* From some experiments which have lately been instituted by MM. Bunsen and Berthold, at Gottingen, it would appear that the hydrated oxide of iron is an antidote to arsenic; but we are sorry to learn that Mr. Brett, who has performed similar experiments in this country, has found the supposed antidote inefficient.

*Creosote.* This substance was discovered by Reichenbach, who procured it first of all from pyroligneous acid, and afterwards from tar. It is antiseptic, as its name implies, which is derived from κρεας, flesh, and σωζω, to preserve. Creosote is also stimulant and caustic, and possesses the property of instantly coagulating albumen; and hence is a styptic. When fresh meat is soaked for an hour in a solution of creosote, and afterwards dried, it may be exposed to the heat of the sun without putrefying; it hardens in about a week, and acquires the smell of good smoked meat, its colour changing to a reddish brown. Fish may be preserved in the same manner; and birds killed by creosote have kept for six weeks without becoming tainted. Dr. Miguet, from whom we have borrowed this account, tells us that he has a bird left which is quite dry, and shows no tendency to putrefaction. Creosote forms two different combinations with water, the first being a solution of one part of creosote in eighty parts of water, and the other a solution of one part of water in ten of creosote.

When pure creosote is applied to the tongue it causes violent pain, while the taste of smoked meat is perceived in the mouth and pharynx, and penetrates into the nasal fossæ; when applied undiluted to the skin it causes a sensation like that produced by a slight burn; the epidermis becomes red, and falls off in small scales.

Plants die if sprinkled with creosote, and a few drops poured on a rose immediately made its colour paler, and soon turned it yellow. A few small fish, some flies, and a spider, were put into two ounces of water, in which a few drops of creosote were suspended; they did not survive more than two minutes. Dr. Miguet remarks, with great justice, that this poisonous action is probably owing to its power of coagulating albumen, and thus destroying the circulation of the blood, by diminishing its fluidity.

A dog two months old took, in the space of a week, eight ounces of distilled water, each containing four drops of creosote. The quantity was doubled during the following week, and produced slow and painful walking, frequent nausea, subsultus tendinum, and occasional tremblings. Another dog was killed by two drachms administered in half an ounce of water. A case of poisoning by creosote is easily distinguishable, as the substances found in the stomach coagulated albumen, and moreover its peculiar odour cannot be mistaken.

Every one has heard of the extravagant panegyrics lavished on *tar-water* by Bishop Berkeley. In one place, he says: "I found all this confirmed by my own experience in the late sickly season of the year 1741, having had twenty-five fevers in my own family cured by this medicinal water drunk copiously. The same method was practised on several of my poor neighbours with equal success. It suddenly calmed the feverish anxieties, and seemed every glass to refresh and infuse life and spirit into the patient. At first some of those patients had been vomited; but afterwards I found that without vomiting, bleeding, blistering, or any other evacuation or medicine whatever, very bad fevers could be cured by the sole drinking of tar-water milkwarm, and in good quantity, perhaps a large glass every hour, taken in bed. And it was remarkable that such as were cured by this comfortable cordial recovered health and spirits at once, while those who had been cured by evacuations often languished long, even after the fever had left them, before they could recover of their medicines, and regain their strength.

"In peripneumonies and pleurisies I have observed tar-water to be excellent, having known some pleuritic persons cured without bleeding, by a blister early applied to the stitch, and the copious drinking of tar-water, four or five quarts, or even more in four and twenty hours. And I do recommend it to farther trial, whether in all cases of a pleurisy, one moderate bleeding, a blister on the spot, and plenty of tepid tar-water may not suffice without those repeated and immoderate bleedings, the bad effects of which are perhaps never got over. I do even suspect that a pleuritic patient betaking himself to bed betimes, and drinking very copiously of tar-water, may be cured by that alone without bleeding, blistering, or any other medicine whatever: certainly I have found this succeed at a glass, every half-hour." (Siris, 2d edit. p. 36.)

And in his second letter to Thomas Prior, esq. the good bishop frankly avows his suspicions that tar-water is a panacea: "I do not say it is a panacea, I only suspect it to be so. Time and trial will shew." It is probably in consequence of these unmerited panegyrics that tar-water has fallen into unmerited neglect; we trust that this may not be the case with the kindred remedy, creosote. We find in Dr. Miguet's work that it has been successfully used externally when diluted, in cases of burns, itch, and other cutaneous diseases, excoriations, gangrene, caries, toothach, whitlow, scrofulous and syphilitic ulcers, &c.; and internally in hemoptysis, and as a fumigation in phthisis, and other pulmonary diseases. We argue better for the fate of creosote than of its elder brother, partly because though extolled in many maladies, it is not called a panacea; and still more because, in the present instance, the eulogies have proceeded from practitioners qualified to understand the diseases they were treating; the tar-water was chiefly employed by the laity. Should the virtues of creosote be confirmed by longer experience, it would be well to return to the use of tar-water, as being not only cheaper but safer.

Creosote may be employed externally, dissolved in eighty parts of water, or a few drops may be poured on a poultice, or in some cases it may be used pure. When used for friction or lotions, from two to six drops are to be dissolved in an ounce of distilled water, or mixed with an ounce of axunge. For internal use two or three drops may be taken three times a day, made up into an emulsion with mucilage and water. To employ it as a fumigation it is only necessary to soak some folds of linen in the creosote, and place them in the apartment of the patient.

*The Ioduret and Hydriodate of Iron.* These preparations have been introduced into practice by Dr. A. T. Thomson, who extols the latter as a remedy of great efficacy in scrofula, chlorosis, and syphilis; and even gives a case of carcinoma which seems to have been cured by it. The solution, he says, is best fitted for medicinal use when each drachm contains three grains of the hydriodate. This quantity may be taken three times a day, as an ordinary dose. A great, and, we fear, a fatal objection to the use of this remedy consists in the extreme facility of its decomposition; besides a host of medicinal substances which decompose it, light, and the small quantity of saline and earthy carbonates contained in common water possess the same power. This would be a matter of comparative indifference if the hydriodate of iron, like many other medicines, became inert by its decomposition; but unfortunately, far from this being the case, when its elements are disunited, one of them, the iodine, becomes poisonously active. We once prescribed the solution of the hydriodate in the dose of a drachm twice a day to a young man, in whom the symptoms of secondary syphilis were fast disappearing. He stayed away a week, and then returned, complaining of the headach, and other troublesome ailments which had been caused by the medicine. In this case we had every reason to believe, from external evidence, that the hydriodate had been decomposed. It will be the duty, therefore, of every one who ventures to prescribe the hydriodate of iron to ascertain its purity, and watch over the first signs of its decomposition with a care which perhaps no other medicine requires.

*Veratria.* This potent alkaloid has of late been extolled as an external application, in an *ex-professo* treatise on the subject, in a strain which has made sober people smile and doubt, and do everything but applaud. If we were to credit the fervid panegyric just mentioned, *(the Currus triumphalis Veratriæ,)* this alkaloid is not merely efficacious in those diseases which are often benefited by external stimulants, but is a panacea in more than half the ills that flesh is heir to, including dropsy, and organic disease of the heart. It is to be exhibited in the form of an ointment, containing twenty grains or more of veratria to the ounce; and though, in the hands of other physicians, this would cause violent excoriation, the vaunter of veratria has never met with this accident. The plain fact appears to be, that veratria like any other unsparing stimulant may be occasionally useful, when applied externally; but unfortunately it is not from the worshipper that we can learn the faults of the idol; nor is it from the writer of the indiscriminating and unscientific treatise in question that we can hope to ascertain the cases in which other and safer stimulants are to be preferred. There is one obvious objection to the general use of veratria—its price. We are informed by Mr. Garden, that this alkaloid, though cheaper than it was, still costs eighteen shillings a drachm, or six guineas an ounce.

*Extract of Artichoke.* This extract has lately been tried in rheumatism, and it is said with some success. It has the recommendation of being harmless, and may therefore be admitted into the endless list of remedies against the perplexing disease for which it is prescribed.

*Smilax Aspera.* This is a species belonging to the same genus as sarsaparilla, and used in the same diseases. The concentrated syrup is sold at exactly one half the price of the concentrated syrup of sarsaparilla,—a consideration of no mean importance. We have observed, or think that we have observed, that, although the recently-introduced syrup is far more aromatic than the old one, yet that, when diluted with water, the old one imparts more flavour. May not a similar practical paradox be observed in some wines when diluted? It seems to us that, though claret has not by itself a stronger taste than port, yet that it imparts more flavour to water.

*New Method of Preparing Prussic Acid.* Mr. Laming has introduced a method of preparing prussic acid, by decomposing the cyanuret of potassium with the bitartrate of potass. We do not know that this possesses any particular advantage over other methods, excepting in the facility which it affords of extemporaneous preparation. Indeed we have been assured, by an eminent practical chemist, that the acid prepared in this manner is not so pure, being always mixed with more or less of the tartrate of potass.

*Lithotrity.* M. Blandin, in his late work, entitled *Parallèle entre la Taille et la Lithotritie,* traces the latter operation as high as Celsus! because the Augustan physician mentions one Ammonius, surnamed ὁ λιθοτομος, not on account of his cutting for the stone, but on account of his cutting the stone itself; but, as Ammonius did not do this excepting when he had attempted lithotomy, and the perineal incision had proved too small, we fear that he can lay but little claim to be the first lithotritist. Perhaps the first person who really performed lithotrity was Gruithuisen, a Bavarian physician, who, in endeavouring to realize the hope which sprung from the labours of Fourcroy and Vauquelin, that stones in the bladder might be chemically dissolved, contrived to drill holes in them, in order to increase the points of contact between them and the solvent. Yet even this was hardly lithotrity, as we now understand the word: the honour of discovering at least the theory of lithotrity must, we think, be given to Mr. Elderton, whose paper on the subject was printed in the Edinburgh Journal for April 1819 (No. lix. p. 261 *et seq.*) He proposed to use a kind of masked file, or, as he calls it, a rasp; and the whole instrument, he says, "bears a strong resemblance to a common, full-sized catheter."

Within the last ten years, the improvements in lithotrity, and lithotritic instruments, have been so numerous, that it would require a volume, rather than the fraction of an essay, to particularize them. We will just mention that the drill and the *percuteur à marteau* are both growing rather obsolete, and Weiss's screw lithotrite is deservedly superseding them.

This instrument is a revival, with improvements, of one invented by Weiss ten years ago. It was tried in 1824 by Sir Benjamin Brodie, on a very hard calculus, the pieces of which broke asunder with so much violence, that he recommended some alteration to be made in the instrument. The spring-saw was accordingly added; but the fact is, that, if the bladder be sufficiently injected, (the great secret in every variety of lithotrity,) the impetus of the fragments need not be feared.

It is remarkable that the application of the screw to lithotrity, which was made ten years since by Mr. Weiss, has lately been claimed by a Mr. L'Estrange, of Dublin, as a recent invention of his own. But Mr. Weiss's instrument stands first, not only in date, but in merit: its superiority to L'Estrange's is so obvious and glaring, as to make a detailed comparison superfluous.

There is one point concerning lithotrity which the impartial critic must never attempt to evade, and that is, that the deaths resulting from this operation are fully as numerous as those following the more dreaded one of lithotomy.

Among other new instruments which we saw a few days since at the shop of the ingenious inventor of the screw lithotrite, we may mention Mr. Shepherd's forceps for the extraction of stumps; Gräfe's apparatus for passing a ligature round polypi, &c.; and a model of a revolving bedstead, invented by Dr. Leo Wolff, of New York. It should rather have been called a revolving bed than bedstead, the object being to turn a patient round together with his bed, the coverlet of the bed being made to support him; and thus the surgeon is enabled to dress or examine the back with the least possible disturbance of the patient. A small umbilical truss, which may be annexed to the busk of the stays, and a spine-splitter, for post-mortem examinations, also seemed to us worthy of note.

*Anatomy and Physiology of the Thymus Gland.* Sir Astley Cooper's work on this subject was published in 1832; but, as productions of this kind have necessarily a very limited circulation, we believe that few of our readers will disapprove of our giving a couple of extracts containing some of Sir Astley's discoveries in the anatomy of the thymus, as well as his opinions on its physiology.

"From what I have said of the structure of the thymus, its composition will be found to be as follows:

"*First.* It is composed of a gland on each side, united only by cellular membrane.

"*Second.* It is formed of two ropes, which can be, with care, unravelled; and they are of considerable length.

"*Third.* The ropes are constituted of small and large lobes, which appear as knots upon the rope.

"*Fourth.* These are disposed in a spiral or serpentine course, from the upper part of the cervical, to the lower extremity of the thoracic portion.

"*Fifth.* Each portion of the rope is a secretory structure.

"*Sixth.* The lobes contain secretory cavities or cells, which may be readily shewn by filling the gland with alcohol, air, gelatin, or even wax.

"*Seventh.* A pouch of communication exists between the lobes and the reservoir.

"*Eighth.* The gland has a central cavity or reservoir.

"*Ninth.* This cavity is not straight, but spiral or serpentine.

"*Tenth.* The reservoir is lined by a very vascular mucous membrane.

"*Eleventh.* The ropes of the gland pass in a spiral or serpentine direction around the mucous membrane, which lines and principally forms the reservoir; and these ropes being united by that membrane to each other, assist in forming the cavity." (P. 32.)

"The office which the thymus is designed to perform is evidently connected with the fœtal stages of existence, as it gradually lessens soon after the child is born; and even when the gland remains of considerable bulk, its secretory cavities are much diminished.

"It has been already stated, that this gland secretes a great abundance of white fluid; that it is situated between the veins in which the great absorbent trunks of the body terminate; that to each cornu is attached a large absorbent duct in the fœtal calf, capable of being filled with coarse injection, and that this vessel terminated at the junction of the jugular veins in the vena innominata." (P. 38.)

"As the thymus secretes all the parts of the blood, viz. albumen, fibrin, and particles, is it not probable that the gland is designed to prepare a fluid well fitted for the fœtal growth and nourishment from the blood of the mother before the birth of the fœtus, and consequently before chyle is formed from food, and this process continues for a short time after birth, the quantity of fluid secreted from the thymus gradually declining as that of chylification becomes perfectly established?" (P. 44.)

This ingenious conjecture had previously been published by Dr. Bow, as we observed at p. 112 of our last number; but we believe that the merit of showing its probability, and almost demonstrating its truth, belongs exclusively to Sir Astley Cooper.

*Bellingeri's Account of the Function of the Nerves.* Bellingeri's physiology of the fifth and seventh pair of nerves has lately been brought forward and advocated by the Edinburgh Medical Journal, as well as by Dr. Negri in the Medical Gazette. As an anatomist, no praise can be too high for Bellengeri; but as a physiologist, though ingenious, he is wrong. He not only attributes both sense and motion to the facial nerves, (an error long since exploded by Mr. Mayo,) but in addition he bestows the same endowments on the seventh; drawing very curious but false distinctions between the supposed endowments of the two. Bellingeri moreover imagines the anterior roots of the spinal nerves to be the source of flexion, and the posterior roots of sensation and extension jointly. Mr. Mayo, to whose letter in the Medical Gazette we are indebted for these particulars, has subjoined the original passages by which they are confirmed. It should be observed, however, that Bellingeri's theories, though now brought forward and discussed for the first time in this country, appeared in Italy in 1818.

*Mr. Kiernan's Anatomy of the Liver* is above all praise, but as our readers will find an abstract of his discoveries in our review of Quain, in this number, it is unnecessary to repeat it here.

*Dr. Carswell's Illustrations of Pathology* conclude an era in the history of morbid anatomy; his book overtops all its contemporaries, and forms the *fluctus decumanus* in the vast ocean of works on the subject. It will be in vain to expect any thing better in the same style; but, if we mistake not, another era is approaching in morbid anatomy, when the most minute structure will be studied, and the very essence of disease will be laid bare before the eye of science.

